# Implication of RAS in Postnatal Cardiac Remodeling, Fibrosis and Dysfunction Induced by Fetal Undernutrition

**DOI:** 10.3390/pathophysiology28020018

**Published:** 2021-06-05

**Authors:** Pilar Rodríguez-Rodríguez, Maria Sofía Vieira-Rocha, Begoña Quintana-Villamandos, Ignacio Monedero-Cobeta, Parichat Prachaney, Angel Luis López de Pablo, Maria del Carmen González, Manuela Morato, Carmen Diniz, Silvia M. Arribas

**Affiliations:** 1 FOSCH Group, Department of Physiology, Faculty of Medicine, Universidad Autónoma de Madrid, 28029 Madrid, Spain; pilar.rodriguezr@uam.es (P.R.-R.); ignacio.monedero@uam.es (I.M.-C.); angel.lopezdepablo@uam.es (A.L.L.d.P.); m.c.gonzalez@uam.es (M.d.C.G.); 2 LAQV/REQUIMTE, Laboratory of Pharmacology, Department of Drug Sciences, Faculty of Pharmacy, University of Porto, 4050-313 Porto, Portugal; mariasofiavieira@gmail.com (M.S.V.-R.); mmorato@ff.up.pt (M.M.); cdiniz@ff.up.pt (C.D.); 3 Department of Anesthesiology, Hospital Universitario Gregorio Marañón, 28009 Madrid, Spain; begoquinti@gmail.com; 4 Department of Pharmacology and Toxicology, Faculty of Medicine, Universidad Complutense de Madrid, 28040 Madrid, Spain; 5 Cardiovascular Research Group, Department of Anatomy, Faculty of Medicine, Khon Kaen University, Khon Kaen 40002, Thailand; parpra@kku.ac.th

**Keywords:** angiotensin II, fetal programming, fibrosis, lactation, left ventricular hypertrophy, cardiovascular remodeling, RAS receptors

## Abstract

Fetal undernutrition is a risk factor for cardiovascular diseases. Male offspring from rats exposed to undernutrition during gestation (MUN) exhibit oxidative stress during perinatal life and develop cardiac dysfunction in ageing. Angiotensin-II is implicated in oxidative stress-mediated cardiovascular fibrosis and remodeling, and lactation is a key developmental window. We aimed to assess if alterations in RAS during lactation participate in cardiac dysfunction associated with fetal undernutrition. Control dams received food *ad libitum*, and MUN had 50% nutrient restriction during the second half of gestation. Both dams were fed *ad libitum* during lactation, and male offspring were studied at weaning. We assessed: ventricular structure and function (echocardiography); blood pressure (intra-arterially, anesthetized rats); collagen content and intramyocardial artery structure (Sirius red, Masson Trichromic); myocardial and intramyocardial artery RAS receptors (immunohistochemistry); plasma angiotensin-II (ELISA) and TGF-β1 protein expression (Western Blot). Compared to Control, MUN offspring exhibited significantly higher plasma Angiotensin-II and a larger left ventricular mass, as well as larger intramyocardial artery media/lumen, interstitial collagen and perivascular collagen. In MUN hearts, TGF-β1 tended to be higher, and the end-diastolic diameter and E/A ratio were significantly lower with no differences in ejection fraction or blood pressure. In the myocardium, no differences between groups were detected in AT1, AT2 or Mas receptors, with MrgD being significantly lower in the MUN group. In intramyocardial arteries from MUN rats, AT1 and Mas receptors were significantly elevated, while AT2 and MrgD were lower compared to Control. **Conclusions**. In rats exposed to fetal undernutrition, RAS disbalance and associated cardiac remodeling during lactation may set the basis for later heart dysfunction.

## 1. Introduction

Epidemiological studies evidence that exposure to an adverse intrauterine environment, such as sub-optimal nutrition, leading to low birth weight (LBW), is associated with increased risk of cardiovascular diseases (CVD), a process known as fetal programing [1]. In animal models of fetal programming induced by various adverse factors, left ventricular hypertrophy (LVH) is frequently found [2,3,4]. Cardiac hypertrophy and remodeling are the structural basis of cardiac dysfunction, ultimately leading to coronary heart disease (CHD) and heart failure.

The perinatal period seems to be a key developmental window where alterations initiated in fetal life may further consolidate, establishing the grounds for disease. Individuals born small usually exhibit accelerated growth during the postnatal period, known as catch-up growth. Studies in LBW children have demonstrated that catch-up growth increases the risk of developing cardio-metabolic diseases later in life [5,6,7]. In experimental animal models, there is also evidence that catch-up growth during lactation has deleterious consequences, leading to adipose tissue accumulation [8,9]. In a rat model of fetal programming induced by undernutrition (MUN rat), we have observed that catch-up growth also affects cardiovascular organs; male rats exposed to undernutrition are born with a smaller aorta, which is enlarged during lactation [10].

Angiotensin II (Ang II) is a well-known factor implicated in cardiovascular remodeling and fibrosis, with some of its effects mediated through oxidative damage [11]. We have evidence that in the perinatal period, undernourished males exhibit an altered oxidative balance, with low levels of antioxidants, elevated plasma biomarkers of oxidative damage [12] and upregulation of cardiac NADPH [13]. Therefore, it is possible that Ang II can participate in the development of early cardiovascular alterations, ultimately leading to hypertension and ventricular dysfunction. The classical RAS involves angiotensin converting enzyme (ACE), Ang II and the AT1/AT2 receptors. AT1 mediates vasoconstriction and proliferation, while AT2 mediates counter-regulatory vasodilator and antiproliferative effects. In addition to the classical axis formed by ACE, accumulating evidence has revealed a new axis, implicating ACE type 2 (ACE2). ACE produces several peptides such as Ang-(1–9), derived from Ang I, alamandine, derived from Ang A and Ang 1(1–7), derived from Ang II or from Ang-(1–9), the latter through the enzymatic activity of ACE or neutral endopeptidase (NEP). In turn, Ang-(1–7) can interact with MasR and Alamandine with MrgD receptors, exerting vasorelaxant, antiproliferative and antifibrotic actions [14], but also exerting a pivotal role in balancing the vasoconstrictor, proliferative and fibrotic actions of Ang II through AT1 receptors [15,16]. These counter-regulatory RAS peptides may have promising actions against cardiac remodeling [17]. Previous clinical and experimental studies have evidenced the implication of RAS alterations in the kidney and vasculature in fetal programming of hypertension [18,19]. Most of the studies have focused on the classical Ang-II axis via AT1 and AT2 receptors. However, there is evidence that the Ang-(1–7) axis, Mas and MrgD receptors, may also contribute to the development and progression of hypertension induced by fetal stress factors [20].

We hypothesize that RAS alterations during lactation may set the basis for later heart disease. To assess this hypothesis, we have studied male offspring from MUN rats, which develop hypertension in adult life and cardiac dysfunction in ageing. At the end of lactation, we assessed: (1) plasma Ang II concentrations; (2) expression of AT1, AT2, Mas and MrgD receptors in the myocardium and intramyocardial arteries, (3) cardiac and intramyocardial artery remodeling and fibrosis and (4) cardiac function. We conclude that in rats exposed to fetal undernutrition, RAS alterations and associated cardiac remodeling along lactation may set the basis for later development of cardiac diseases.

## 2. Materials and Methods

### 2.1. Maternal Undernutrition (MUN) Model

Experiments were performed in Sprague Dawley rats from the colony maintained at the Animal House facility of the Universidad Autónoma de Madrid (ES-28079-0000097). All animal procedures were performed according to the guidelines from Directive 2010/63/EU on the protection of animals for scientific purposes, from the Spanish legislation (RD 1201/2005), and they were approved by the Ethics Review Boards of Universidad Autónoma de Madrid (CEI-UAM 96-1776-A286) and the Regional Environment Committee of the Comunidad Autónoma de Madrid (RD 53/2013; Ref. PROEX 04/19; date 19 March 2019).

The rats were housed in buckets 36.5/21.5/18.5 cm (length/width/height) on aspen wood bedding and maintained under controlled conditions (temperature 22 °C, relative humidity 40% and 12/12 light/dark photoperiod). They were fed with a breeding diet (EuroRodent Diet 22; 5LF5, Labdiet, Madrid, Spain) containing 55% carbohydrates, 22% protein, 4.4% fat, 4.1% fiber and 5.4% mineral, with a sodium content of 0.26%. Drinking water was provided ad libitum in all cases.

The experimental model was induced as previously described [13]. Twelve-week-old female rats were mated, and day 1 of gestation was determined by observation of sperm in the vaginal smear. Thereafter, they were randomly allocated to the Control group (C, *n* = 5 dams) or undernutrition group (MUN, *n* = 5 dams). C dams received the *ad libitum* diet throughout pregnancy, and MUN dams received the *ad libitum* diet during the first 10 days of gestation, but only 50% of the usual daily intake from day 11 to the end of pregnancy. After delivery, both C and MUN dams were offered food *ad libitum* during the whole lactation period. After birth, the pups were sexed, and the litter was standardized to 12 individuals (smaller litters were not used).

### 2.2. Experimental Protocols

All experimental procedures were performed at weaning in male offspring from C and MUN groups. The experiments were performed in 1–2 males from each litter, and data were averaged, considering sample size the number of litters, following the recommendations for studies in experimental models of fetal programming [21]. The remaining animals from each litter were used for other studies according to the 3Rs rule for experimental animals [22].

The experimental procedures were organized as follows. The rats were weighted at birth and at weaning (age 21 days). At the age of 21 days, echocardiography was performed under anesthesia (for details, see next section). Thereafter, the rats were allowed to recover for a period of 1–2 days. Then, hemodynamic parameters were measured under anesthesia (for details, see next sections), and, at the end of the recording period, a blood sample was collected in EDTA tubes. Thereafter, the animals were killed by exsanguination, and the heart and tibia were dissected.

The blood sample was centrifuged at 1000 g for 10 min at 4 °C to obtain plasma, which was stored at −80 °C until use for quantification of Ang II.

The heart was rinsed with saline; the atria were discarded; and the ventricles were weighted with an analytical balance (Boeco, Hamburg, Germany) fixed in 4% paraformaldehyde (PFA) and paraffin-embedded for histological procedures. The tibia was measured with a digital caliper (Comecta, Nessler, Madrid, Spain) to quantify heart weight relative to tibia length. Some hearts were snap frozen and kept at −80 °C for analysis of protein expression by Western blot.

### 2.3. Transthoracic Echocardiography (TTE)

TTE was performed as previously described [13,23]. Briefly, the rats were anesthetized with diazepam 4 mg/kg and ketamine 10 mg/kg i.p. TTE was performed using the VIVID q system (GE Healthcare, Munich, Germany) equipped with a 13-MHz probe (12S-RS, GE). The images were acquired with the animals in the left lateral decubitus. M-mode imaging of the parasternal short-axis (papillary level) view allowed measurement of the end-diastolic internal diameter (LVIDd) and end-systolic internal diameter (LVIDs), posterior wall thickness at diastole (PWd) and interventricular septum thickness at diastole (IVSd). Values were determined by averaging the measurements of 3 consecutive cardiac cycles in accordance with the American Society of Echocardiography guidelines [24]. The above measurements were used to calculate left ventricular mass (LVM), expressed in grams, as previously described [25], using the following Equation (1):

LVM = 0.8 [1.04 (IVSd + LVIDd + PWd)3 − (LVIDd)3] + 0.6 g(1)

Echocardiographic parameters were calculated as previously described [23]. LVM was adjusted for body weight by calculating the left ventricular mass index (LVMI). Left ventricular ejection fraction (LVEF) and fractional shortening were calculated as measures of LV systolic function. The pulsed-wave Doppler early-to-late transmitral peak diastolic flow velocity ratio (E/A ratio) was calculated to assess diastolic function (E, mitral peak early-filling velocity; and A, mitral peak flow velocity at atrial contraction). The transmitral flow velocity profile was determined by positioning a sample volume at the tip of the mitral valve on the para-apical long-axis view. The E-wave deceleration time was measured as the time interval between peak E-wave velocity and the point where the descending E-wave (or its extrapolation) intercepted the zero line. Relative parietal wall thickness (PWT) was calculated as IVSd + PWd/LVIDd.

### 2.4. Hemodynamic Parameters

Briefly, as previously described [13], the rats were anaesthetized with 37.5 mg/kg ketamine hydrochloride and 0.25 mg/kg medetomidine hydrochloride i.p. and placed on a heating blanket. The iliac artery was exposed, and a catheter was inserted (filled with 0.9% saline solution with 1% heparin). The catheter was connected to a pressure transducer (Statham; Harvard Apparatus, Holliston, MA, USA) and to a PowerLab system/8SP (ADInstruments; Oxford, UK). The pressure wave was displayed for approximately 45 min and stored for later analysis of data. Heart rate (HR) and systolic and diastolic blood pressure (SBP, DBP) were measured in the chart, averaging over approximately 1 min of the last part of the recording period.

### 2.5. Histology

Serial 5 µm thick sections were cut from the paraffin-embedded ventricles below the chordae tendineae with a microtome (Leitz, Wetzlar, Germany). Thereafter, they were dewaxed, rehydrated, and used for several histological and immunohistochemical procedures, as describe below. Morphometry was performed with FIJI software [26].

To assess myocardial wall area, heart sections were stained with hematoxylin-eosin. Sections were visualized with a ×10 objective using an Olympus DMLB microscope, and images were taken with a high-resolution Leica DC200 digital camera (Leica Microsystems, Heerbrugg, Switzerland). From each image, left and right ventricular areas were separately quantified. Data were expressed as mm^2^.

To analyze intramyocardial artery structure, 4–5 images from each left ventricle were acquired with a ×40 objective. In every image, all intramyocardial arteries were measured, analyzing total artery and lumen areas. From these parameters, the wall cross sectional area was calculated as total artery area – lumen area. Since there were arteries with different sizes, the relative media/lumen areas were calculated to normalize the data.

To quantify collagen content, sections were stained with Sirius red [27] and visualized with an Olympus DMLB microscope, equipped with a polarizer/analyzer set, using a ×10 or a ×40 objective, for the myocardium and intramyocardial arteries, respectively.

Polarized light images were subsequently transformed to binary images with FIJI software. The images were first thresholded, and binary images were obtained. Collagen relative content in the myocardium was quantified as the collagen-stained area relative to total myocardial area. From each ventricle, 5 different regions were quantified, and their relative collagen areas averaged. Intramyocardial artery collagen was evaluated as the collagen-stained area relative to lumen area.

### 2.6. Immunohistochemistry

Immunohistochemistry was performed as previously described [28] with some modifications. Briefly, sections were incubated with rabbit primary polyclonal antibodies (1:50 dilution; overnight; 4 °C, in a humidified chamber). The following primary antibodies were used: anti-AT1 (Santa Cruz biotechnology: sc-31,181; Heidelberg, Gernany), anti-AT2 (Abcam: ab19134; Cambridge, UK), anti-Mas (Abcam: ab197992; Cambridge, UK) and anti-MrgD (Alomone Labs: AMR-061; Jerusalem, Israel). The specificity of the primary antibodies has been established in previous studies; AT1 [29,30]; AT2 [31,32]; Mas [33] and MrgD [34]. In addition, specificity for all the primary antibodies was tested in our experimental conditions, by pre-adsorbing the individual primary antibody with a tenfold excess of its respective blocking peptides, overnight, at 4 °C (data not shown). Sections were incubated using the avidin-biotin complex (ABC) and 3,3’-diaminobenzidine tetrahydrochloride (DAB) as a chromogen. For negative controls (controls for non-specific binding of the secondary antibody), primary antibodies were omitted (data not shown). Micrographs of each immunostained section were acquired using a CDC camera (Leica DFC295, Leica Microsystems, Heerbrugg, Switzerland) mounted on the microscope Nikon Eclipse E400 (Nikon Corporation, Tokyo, Japan) with a ×20 objective (0.5 NA, cover glass thickness, 0.17 mm; working distance 2.1;), using Leica Microsystems software version 3.5.0 (Leica Microsystems, Heerbrugg, Switzerland). Illumination conditions of the bright field optics and camera exposure were maintained constant throughout the acquisition of all tissue sections, including negative control sections. Acquired images (24 bit, 8 bits/color), with a resolution of 3072 × 2304 pixels, corresponded to a 655.36 × 491.52 µm area on the original histological section (1 pixel = 0.21 µm; a calibration micrometer slide was used to convert pixels into µm).

Histomorphometric analysis has been previously described as a valid methodology [35,36,37]. Quantitative analysis/processing of digital images from DAB-immunostained sections were assessed using the SACAIA method and the PAQI software (CEMUP, Porto, Portugal), as previously described [38]. Briefly, from RGB (red, green, blue) digital color images, only the blue component was selected for analysis, due to its higher contrast. Boundaries were delineated to extract the object of interest and to set thresholds for automated DAB-staining segmentation using image analysis. As immunohistochemistry can provide detailed information concerning the location/presence/area of immunostaining, to make the analysis more comprehensive, we evaluated the expression on two different cardiac structures: intramyocardial arteries and the myocardium. To determine differences between stained and non-stained tissue, negative control sections were imaged with the same microscope-illumination and camera-operating conditions, and the average of stained level was determined: a value of 171 for a maximum of 255. This average value was used for threshold segmentation of the stained areas of each structure. The level of immunostaining was obtained by quantifying the fraction of the tissue that was stained with DAB (stained fractional area) using digital images of DAB-labeled immunostains from heart sections.

### 2.7. Quantification of Plasma Ang II Levels

Ang II was extracted from plasma samples by solid phase extraction (Discovery^®^ DSC-Ph SPE tube (Sigma-Aldrich, Lisbon, Portugal), and Ang II levels were quantified by ELISA using a commercial kit (Peninsula Laboratories International).

### 2.8. Western Blot

Snap frozen hearts were used to quantify protein expression of TGF-β1 as previously described [13]. Fifty µg protein samples were separated by 10% SDS-PAGE gels, and the primary antibody against TGF-β1 (rabbit polyclonal Santa Cruz Biotechnology; 1:1000 dilution; Heidelberg, Germany) was applied overnight at 4 °C. Thereafter, the primary antibody was washed, and the secondary antibody (anti-rabbit IgG-peroxidase conjugated, dilution 1:10000) was applied for 1 h at 37 °C. The blots were then washed and incubated in commercial enhanced chemiluminescence reagents (ECL Prime, Amersham Biosciences, Buckinghamshire, UK), and the bands were detected by the ChemiDoc XRS + Imaging System (Bio-Rad, Hércules, CA, USA). To prove equal loadings of samples, blots were re-incubated with the GADPH antibody (1:3000 dilution; Sigma-Aldrich, Hércules, CA, USA). Blots were quantified using Image Lab 6.1 software (Bio-Rad, Hércules, CA, USA), and expression values were normalized with GADPH.

### 2.9. Statistical Analysis

Statistical analysis was performed with GraphPad Prism (version 5, San Diego, CA, USA). The Kolmogorov–Smirnov test was used to analyze the normality of the data. When more than one animal per litter was used, the data were averaged, and sample size was considered the number of different litters. Data followed a normal distribution; therefore, they were expressed as mean ± standard error of mean (SEM). Student’s t test was used to assess statistical differences between C and MUN offspring. A statistically significant level was established at *p* < 0.05.

## 3. Results

### 3.1. Anthropometric Variables

At birth, body weight was significantly smaller in MUN male rats compared to Control (MUN = 4.62± 0.24 g, *n* = 30 rats, 5 litters; C = 6.71± 0.25 g, *n* = 30 rats, 5 litters; *p* < 0.05). At weaning there was no significant difference between MUN and control rats either in body weight (MUN = 50.01 ± 2.6 g; *n* = 7 rats, 5 litters; C = 52.4 ± 1.7 g; *p*-value = 0.31) or tibial length (MUN = 20.33 ±0.3 mm; C = 20.55 ±0.40 mm; *n* = 7 rats, 5 litters, *p*-value = 0.61).

### 3.2. Hemodynamic Parameters

No significant differences were detected between Control and MUN rats in SBP (Control = 85.99 ± 4.18 mmHg, *n* = 10 rats, 5 litters; MUN = 79.22 ± 6.53 mmHg, *n* = 10 rats, 5 litters), DBP (Control = 43.59 ± 2.05 mmHg, *n* = 10 rats, 5 litters; MUN = 37.61 ± 5.05 mmHg, *n* = 10 rats, 5 litters) or HR (Control = 308 ± 26 bpm, *n* = 10 rats, 5 litters; MUN = 319 ± 18 bpm, *n* = 10 rats, 5 litters).

### 3.3. Transthoracic Echocardiography

**Figure 1:**
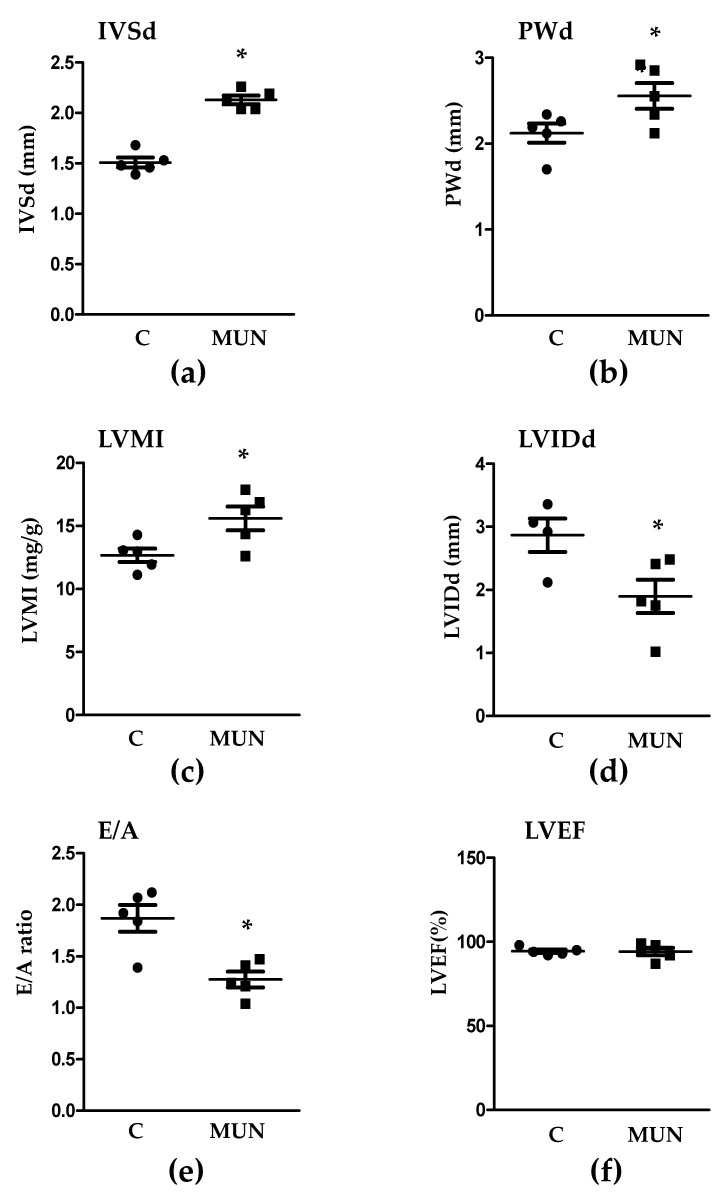
Echocardiographic parameters in 21-day old male offspring from rats exposed to maternal undernutrition during pregnancy (MUN) and control rats fed ad libitum (C). (**a**) IVSd, interventricular septum thickness at diastole; (**b**) PWd, posterior wall thickness at diastole; (**c**) LVMI, left ventricular mass index; (**d**) LVIDd, left ventricular internal diameter at diastole; (**e**) E/A, E, mitral peak early-filling velocity, and A, mitral peak flow velocity at atrial contraction; (**f**) LVEF, left ventricular ejection fraction. Sample size per group: 6 rats from 5 litters. Student’s t test, * *p* < 0.05 compared to C rats.

MUN males showed a significantly larger IVSd, PWd and LVMI compared to Controls (Figure 1). The LVIDd and E/A ratio were significantly smaller in MUN rats when compared to Control. No statistical differences were detected in LVEF (Figure 1).

### 3.4. Heart and Intramyocardial Artery Morphology

Compared to Control, MUN offspring exhibited a significantly larger heart weight/body weight (C = 5.1 ± 0.1 mg/g; *n* = 10 rats, 5 litters; MUN = 5.9 ± 0.0 mg/g; *n* = 10 rats, 5 litters, *p* < 0.05), and heart weight/tibial length (C = 11.9 ± 0.2 mg/mm; *n* = 10 rats, 5 litters; MUN = 14.1 ± 0.9 mg/mm, *n* = 10 rats, 5 litters).

**Figure 2:**
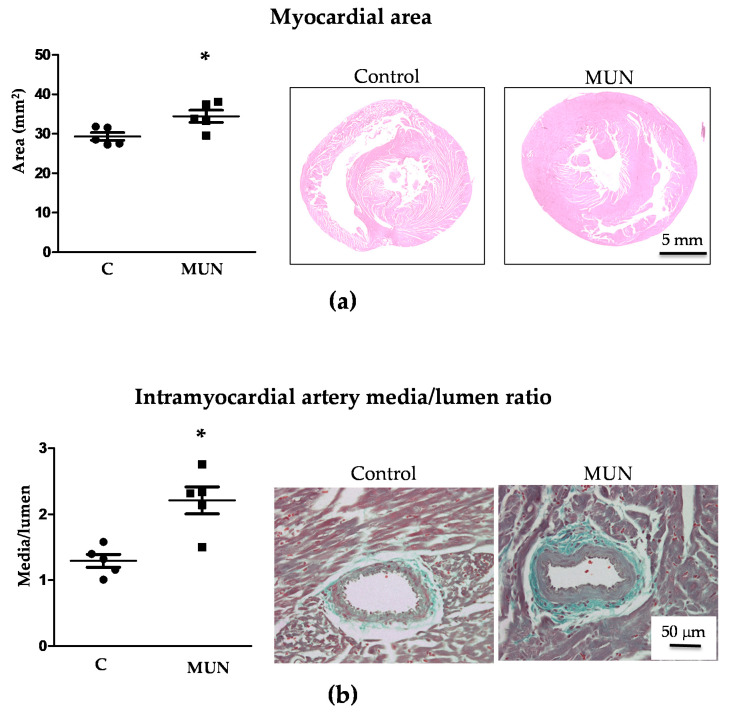
Myocardial and intramyocardial artery area in 21-day old male offspring from rats exposed to maternal undernutrition during pregnancy (MUN) and control rats fed ad libitum (C). (**a**) Myocardial area with representative images from sections stained with hematoxylin-eosin and captured with ×10 objective; (**b**) intramyocardial artery media/lumen and representative images stained with Masson Trichrome Stain and captured with a ×40 objective. Sample size per group: 7 rats from 5 litters. Student’s t test, * *p* < 0.05 compared to C rats.

Histological analysis revealed that myocardial area was also significantly larger in MUN compared to C rats (Figure 2), both the area of left ventricle (C = 23.6 ± 0.5, MUN = 26.9 ± 1.0 mm^2^, *p* < 0.05) and right ventricle (C = 5.7 ± 0.4, MUN = 8.7 ± 0.3 mm, *p* < 0.01).

In intramyocardial arteries, the relative media/lumen area was also significantly larger in MUN compared to C rats (Figure 2).

**Figure 3:**
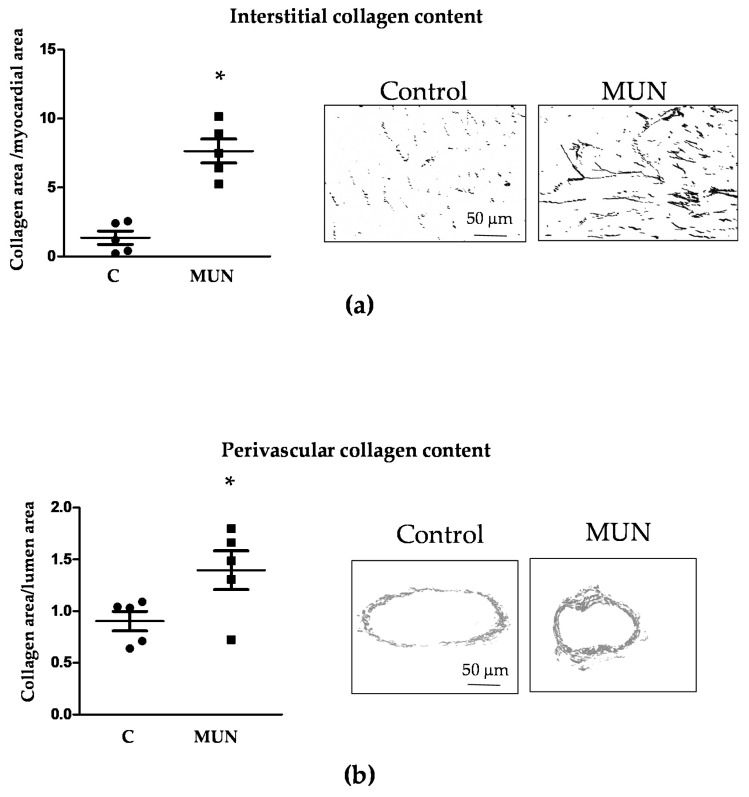
Heart collagen content in 21-day old male offspring from rats exposed to maternal undernutrition during pregnancy (MUN) and control rats fed ad libitum (C). (**a**) Interstitial collagen and representative binary images; (**b**) perivascular intramyocardial artery collagen and representative binary images. Heart sections were stained with Sirius red; images were obtained at ×10 (myocardium) or ×40 objective (intramyocardial arteries) and transformed in binary images for quantification. Left panels show quantitative analysis of the relative area occupied by collagen. Sample size per group: 7 rats from 5 litters. Student’s t test, * *p* < 0.05 compared to C rats.

Interstitial collagen content was significantly larger in the ventricles from MUN rats compared to Controls. Perivascular collagen content was also significantly higher in intramyocardial arteries from MUN compared to Control rats (Figure 3).

### 3.5. Expression Of RAS Receptors in Myocardium and Intramyocardial Arteries

**Figure 4:**
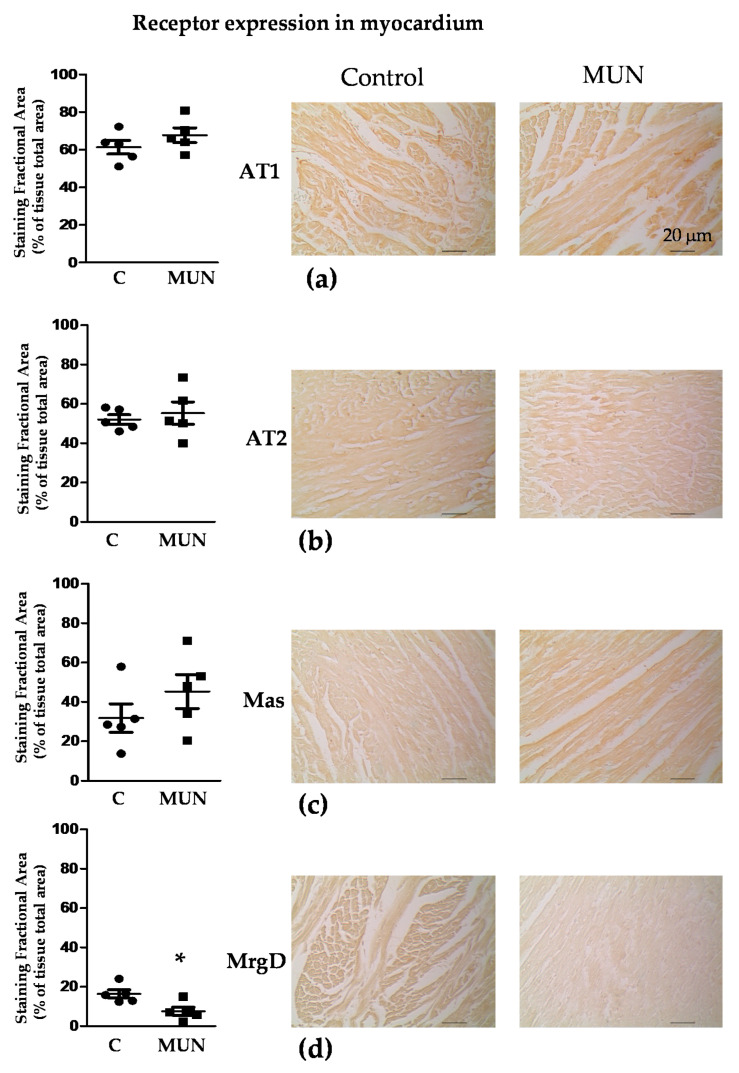
Immunohistochemistry for RAS receptors in the myocardium from 21-day old male offspring from rats exposed to maternal undernutrition during gestation (MUN), and control rats fed ad libitum (C). (**a**) AT1 receptors; (**b**) AT2 receptors; (**c**) Mas receptors; (**d**) MrgD receptors. Heart sections were stained with individual primary antibodies; the resulting immuno-complexes were detected with a biotinylated secondary antibody and amplified by ABC complex, using 3,3′-diaminobenzidine (DAB) as chromogen. Images were obtained with a ×20 objective. Left panels show quantitative analysis of MUN and C myocardium-stained fractional areas (percentage of tissue total area) using SACAIA method. Representative DAB images are shown in right panels (scale bar = 20 μm). Sample size per group: 7 rats from 5 litters. Student’s t test, * *p* < 0.05 compared to C.

Immunoreactivity against the AT1, AT2, Mas and MrgD receptors was observed in the myocardium from MUN and C rats. No statistical differences between C and MUN rats were detected in AT1, AT2 or Mas receptors, while there was a lower expression of MrgD receptors in MUN rats compared to C (Figure 4).

**Figure 5:**
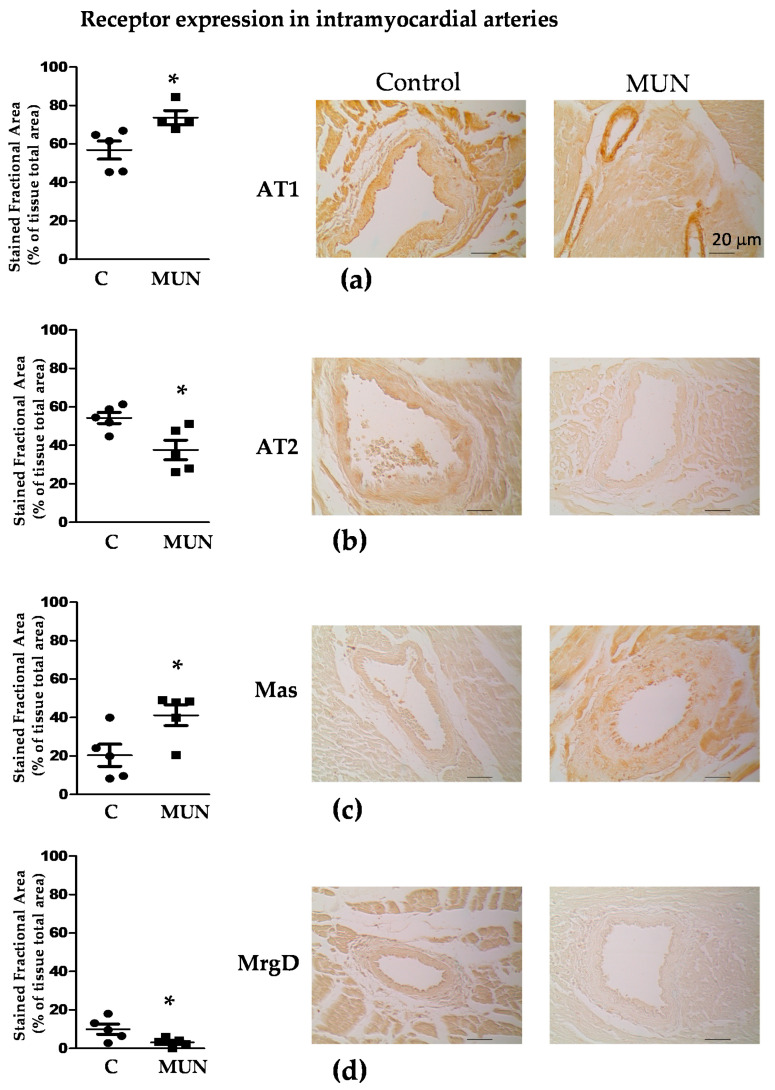
Immunohistochemistry for RAS receptors in intramyocardial arteries from 21-day old male offspring from rats exposed to maternal undernutrition during pregnancy (MUN) and control rats fed ad libitum (C). (**a**) AT1 receptors; (**b**) AT2 receptors; (**c**) Mas receptors; (**d**) MrgD receptors. Heart sections were stained with individual primary antibodies; the resulting immunocomplexes were detected with a biotinylated secondary antibody, and amplified by ABC complex, using 3,3′-diaminobenzidine (DAB) as chromogen. Images were obtained with a ×20 objective. Left panels show quantitative analysis of MUN and C intramyocardial artery-stained fractional areas (percentage of tissue total area) using SACAIA method. Representative DAB images are shown in right panels (scale bar = 20 μm). Sample size per group: 7 rats from 5 litters. Student’s t test, * *p* < 0.05 compared to C rats.

All receptor types were expressed in intramyocardial arteries, being more abundant in the intima than in the media and adventitial layers. In C rats, immunoreactivities for AT1 and AT2 receptors were higher than for Mas and MrgD receptors. In MUN rats, AT1 presented the highest expression, followed by similar immunoreactivities of AT2 and Mas receptors, with the MrgD receptor having the lowest immunoreactivity. Intramyocardial arteries from MUN rats exhibited a significantly higher expression of AT1 and Mas receptors than controls. By contrast, AT2 and MrgD receptor expression was lower in MUN when compared to C rats (Figure 5).

### 3.6. Plasma Ang II Levels

Plasma concentration of Ang II was markedly higher in MUN rats compared to Controls (Control = 0.87 ± 0.38 pg/mL, *n* = 5 rats, 5 litters; MUN = 59.42 ± 17.70 pg/mL, *n* = 7 rats, 5; litters; *p* < 0.05). We also detected that 50% of Control rats had plasma Ang II levels below the detection level of the ELISA kit, while in the MUN group, only 15% of the rats were below detection level.

### 3.7. TGF-β1 Protein Expression

**Figure 6:**
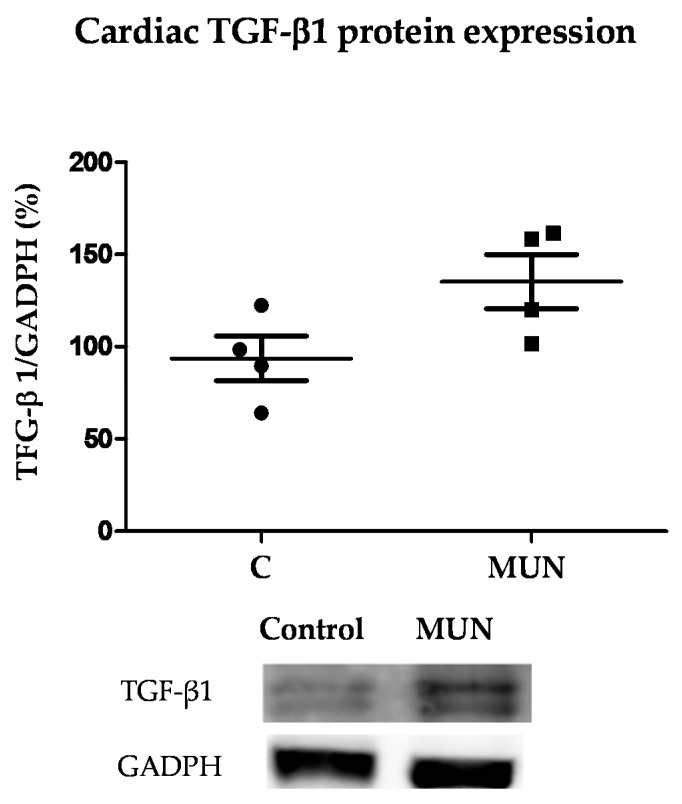
Protein expression level of TGF-β1 by Western blot in cardiac tissue from 21-day old male rats. Results show the densitometric analysis, relativized to GADPH expression and representative examples. Sample size per group: 5–7 rats from 4 litters. Student’s *t* test.

The protein expression level of TGF-β1 tended to be higher in ventricles from MUN rats but did not reach a statistically significant difference (*p* = 0.070) (Figure 6).

## 4. Discussion

This work aimed to assess if dysregulation of Ang II and RAS receptors in rats exposed to insufficient nutrient supply in utero is associated with cardiac alterations during lactation, which may set the basis for the reported ventricular dysfunction in this animal model of fetal programming in ageing.

In the present study, we found that MUN males exhibit LVH at weaning, evidenced by the larger relative heart weight, which was also confirmed by histology and echocardiography. Our findings are in accordance with data reported in other animal models of fetal programming [2,3,39]. We have previously shown—and confirmed in the present study—that MUN offspring are born with lower weight compared to Control rats, but exhibit a quicker weight gain during lactation, reaching a similar body weight by weaning. Our previous reports evidenced a disproportionate growth of cardiovascular tissues during this rapid growth period. Thus, the aorta of MUN rats, that exhibits hypotrophy at birth, develops hypertrophic remodeling by weaning [10]. Similarly, we have evidence that, at birth, the heart weight/body weight ratio is significantly reduced in MUN male rats when compared to sex-matched counterparts, and, in the present study, we evidenced enlarged ventricular mass at the end of lactation. Our results are in accordance with those observed in rats exposed to protein restriction during fetal life, i.e., they are born with a thinner left ventricular wall, and, during lactation, they have a steeper cardiac growth, resulting in hypertrophy around 28 days of postnatal life. Taken together, these data suggest that lactation is a critical developmental window, when a disproportionate cardiovascular growth may set the basis for CVD later in life.

As expected, in MUN rats we also detected hypertrophic remodeling of intramyocardial arteries. Coronary artery enlargement has also been described in male rats exposed to Zn restriction during the intrauterine period. However, in this rat model, coronary artery hypertrophy was shown to be associated with hypertension [40], which is a well-known stimulus for cardiac and vascular remodeling. We discarded hypertension as an initiating factor, since, at the age of 21 days, MUN males exhibited similar blood pressure levels compared to Control rats.

Ang II is known to exert trophic actions in the cardiovascular system [11], being associated with CVD. We detected a significant elevation of Ang II in the plasma of MUN males at weaning, which may account for the perinatal hypertrophy found in heart in the present study, and previously reported in blood vessels [10]. The implication of RAS in both renal and vascular systems, and its effects on the development of hypertension, has been widely demonstrated in several models of fetal programming, [19]. RAS alterations in early stages of life seem to contribute to later development of CVD in other models of hypertension. In spontaneously hypertensive rats (SHR), ACE inhibition during gestation is able to reprogram the development of hypertension and LVH in the offspring, leading to an attenuated form of the disease [41]. Similarly, treatment of SHR dams with the ACE inhibitor captopril reduced heart hypertrophy, fibrosis, and vascular remodeling in the offspring [42]. Furthermore, in a mice model of fetal programming induced by maternal undernutrition, there is evidence of increased angiotensinogen and ACE mRNA expression in fetal hearts, associated with later development of LVH [43]. The markedly higher plasma levels of Ang-II in MUN compared to Control rats suggests that the ACE/Ang II axis predominates over the ACE-2/Ang-(1–7) and Ang-(1–9) axis. This is in accordance with data reported in sheep exposed to glucocorticoids during gestation, which evidenced a lower ACE2 activity in this model of fetal programming [20]. These data suggest that an excess in Ang II circulating levels may be a common alteration induced by exposure to different fetal stress factors.

Fibrosis, together with inflammation and cell growth, are one of the characteristic pathological features of an elevated Ang II. In the present study, we found an increased collagen content in both the myocardium and in the adventitia of intramyocardial arteries from MUN rats. TGF-β1 is one of the factors implicated in cardiac hypertrophy and fibrosis, which is upregulated in response to several pathological stimuli [44], including excess Ang II [45]. We found a higher, but not significant, elevation of TGF-β1 in MUN hearts. Therefore, although it is possible that it can play a role in the observed cardiac remodeling, it may not be the sole mechanism. Several cytokines associated with cardiac hypertrophy are also synthesized by fibroblasts and cardiomyocytes, as well as by circulating immune cells [44], and it has been proposed that low grade of systemic inflammation leading to oxidative stress may contribute to myocardial remodeling. For example, in patients with preserved ejection fraction, this is a plausible mechanism leading to cardiomyocyte hypertrophy [46]. In addition, in a rat model of fetal programming induced by preeclampsia, myocardial inflammatory pathways have been described in males, but not in females [47]. 21-day old MUN males exhibited a normal ejection fraction, but diastolic dysfunction together with hypertrophy and fibrosis, and we have previously demonstrated that males, but not females, exhibit increased plasma oxidative damage biomarkers [12] and elevated cardiac NADPH oxidase expression [13]. Since oxidative stress plays a central element in cardiac hypertrophy and fibrosis in the context of elevated Ang II, through the activation of NADPH oxidase [44,48], we suggest that oxidative damage could be a potential mechanism implicated in the Ang-II mediated pathological responses observed in the heart from MUN males. The role of inflammation as intermediate mechanisms between fetal undernutrition and cardiovascular remodeling deserves additional studies.

Regarding the contribution of the four types of receptors analyzed, in intramyocardial arteries from MUN rats, we found a higher immunoreactivity of AT1, but a lower expression of AT2 receptors. Regarding the contribution of receptors for Ang-(1–7) and alamandine, we found an increase in Mas, but a decrease in the MrgD receptor. Both receptors play a similar role, reducing fibrosis and increasing vasodilatation [15,49]. Since the highest immunoreactivity was found for AT1 receptors, we suggest that AT1-mediated responses are likely to play a major role in the intramyocardial artery remodeling process. This could also explain our previous findings regarding oxidative stress in this rat model, since NADPH oxidase effects are mediated through AT1 receptors [50]. The lower expression of AT2 and MrgD receptors may also participate, through a reduction in their antifibrotic and antiproliferative counter-regulatory actions. Besides, a reduction in these receptors could contribute to coronary artery dysfunction later in life by reducing vascular relaxation. In the myocardium from MUN rats, we did not find a significant difference in AT1, AT2 or Mas receptors, but did find a lower MrgD immunoreactivity. This receptor counteracts the proliferative and fibrotic actions of Ang II [28,38]. AT1 exhibited the highest immunoreactivity from all the receptors analyzed, and Ang II was markedly elevated in the plasma from MUN rats. We suggest that a reduced expression of MrgD could result in a disbalance between proliferative/antiproliferative responses, contributing to the observed ventricular hypertrophy.

Echocardiographic data showed that, by the age of 21 days, MUN males already exhibited a reduction in diastolic function, demonstrated by the significant decrease in the E/A ratio. This alteration is likely the consequence of cardiac remodeling. The elevation of LVM and the increased PWT are indicative of concentric remodeling [51]. This structural alteration, together with the elevation of interstitial collagen, increased ventricular stiffness, reducing the filling capacity of the ventricles, as demonstrated by the smaller end-diastolic diameter in the hearts from MUN rats. Despite the alteration in diastolic function, we did not find significant changes in the ejection fraction, which we have reported in MUN rats at old age [13]. Cardiac dysfunction in adult and old age has been previously described in other animal models of fetal programming. For example, moderate undernutrition during intrauterine life in baboons produces myocardial remodeling and reduced cardiac function in adults [52]. Rats exposed to hypoxia or to undernutrition during fetal life showed increased left ventricular end diastolic pressure and reduced recovery after ischemia reperfusion injury at the age of 7 months [4]. However, early diastolic dysfunction induced by fetal programming has not been previously reported. The presence of concentric remodeling is associated with a worse prognosis and heart failure [23]. Therefore, our data suggest that the perinatal development of concentric remodeling and diastolic dysfunction in MUN rats represents an initial stage of heart disease, developing into heart failure in ageing. Besides, a rigid ventricle, together with the observed intramyocardial artery remodeling and fibrosis, may also compromise coronary artery function.

ACE inhibitors and AT1-blockers are widely prescribed for the treatment of hypertension and congestive heart failure in humans. In animal models of fetal programming, these drugs have been shown to be effective in blood pressure lowering [19]. Our data add evidence that early RAS alterations may be an initial step in later development of heart failure and suggest the possibility to prevent cardiac dysfunction by targeting RAS. Further studies are needed to analyze the potential of RAS blockade in early stages of life as a therapeutic target in LBW individuals with high risk of CVD.

## 5. Conclusions

**Figure 7:**
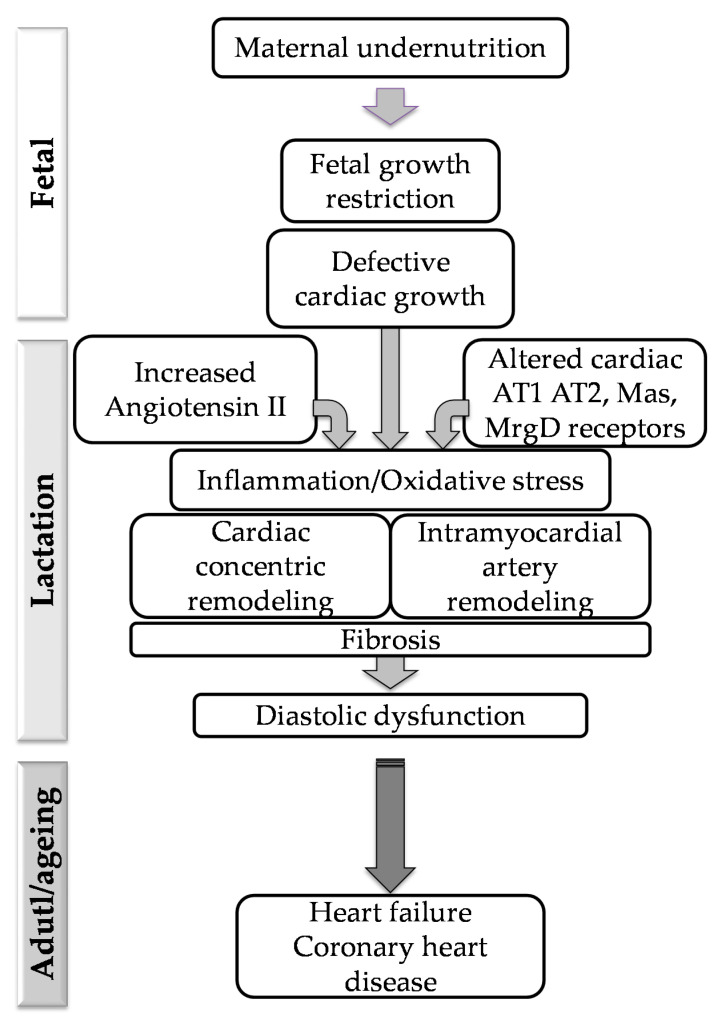
Schematic diagram depicting the relationship between fetal undernutrition, alterations in Ang II and RAS cardiac receptors during lactation and development of cardiac structural and functional alterations, which may contribute to long-term heart disease.

We found that fetal undernutrition followed by accelerated growth induces cardiac concentric hypertrophy and intramyocardial artery remodeling, accompanied by fibrosis, together with alterations in Ang II levels and a disbalance in some RAS receptors. We suggest that the aforementioned alterations may be responsible for the reduced diastolic function already observed at this early age, which can set the basis for later development of cardiac dysfunction and coronary heart disease in later stages of life. Based on our previous data evidencing increased ROS and NADPH oxidase expression in the heart during lactation, we suggest that oxidative stress plays a central role and may be the link between RAS disequilibrium and cardiac structural alterations, and the role of inflammation as a mediator of these responses deserves further attention. Figure 7 summarizes the main conclusions of the study.

## Data Availability

Raw data can be provided upon request to the corresponding author.
